# Research on a Micro-Processing Technology for Fabricating Complex Structures in Single-Crystal Quartz

**DOI:** 10.3390/mi11030337

**Published:** 2020-03-24

**Authors:** Chao Han, Cun Li, Yulong Zhao, Bo Li, Xueyong Wei

**Affiliations:** State Key Laboratory for Manufacturing Systems Engineering, Xi’an Jiaotong University, Xi’an 710049, China; hc2113@stu.xjtu.edu.cn (C.H.); zhaoyulong@mail.xjtu.edu.cn (Y.Z.); li.bo.123.666@stu.xjtu.edu.cn (B.L.); seanwei@xjtu.edu.cn (X.W.)

**Keywords:** quartz on silicon (QoS), bonding, ICP etching

## Abstract

Single-crystal quartz material is widely applied in the manufacture of resonators and sensors, but it is difficult to process because of its high hardness. A novel way to fabricate single-crystal quartz structures is proposed in this paper; the method includes quartz-on-silicon (QoS) technology and inductively coupled plasma (ICP) etching, which makes it feasible to fabricate complex structures with crystal quartz. The QoS method encompasses the bonding of silicon and quartz, followed by the thinning and polishing of quartz, which can enable the fabrication of an ultra-thin quartz wafer on silicon. In this way, instead of the conventional wet etching with hydrofluoric acid, the quartz layer can be easily etched using the ICP dry-etching method. Then, the structure of the pure quartz material is obtained by removing the silicon wafer. In addition, the silicon layer can be processed into the appropriate structure. This aspect overcomes the difficulty of processing a complex structure of single-crystal quartz with different crystal orientations. Thin single-crystal quartz wafers of Z-cut with a thickness of less than 40 μm were obtained by using this method, and a complex three-dimensional structure with an 80 μm width was also acquired by the ICP etching of the quartz wafer. The method can be applied to make both crystal-oriented quartz-based sensors and actuators, such as quartz resonant accelerometers.

## 1. Introduction

Single-crystal quartz material has captured significant attention, which is attributed to its outstanding material properties in sensor manufacturing [1,2]. Quartz crystal not only possesses the piezoelectric property, but also has excellent mechanical, electrical, and temperature characteristics. It is designed and manufactured for resonators, oscillators, and filters because of its exceptional performance in frequency stabilization and frequency selection [3,4]. Furthermore, the piezoelectricity of quartz material can be used to excite the resonators into vibration to simplify both sensor structures and excitation circuits [5].

The strong covalent bond characteristic of single-crystal quartz material makes it difficult to alter, and its high hardness and difficult processing characteristics also impede the improvement of device performance. The traditional methods used to process quartz structures mainly include fluoride-based wet etching [6], laser micro-/nano-processing (LMP) [7,8], and inductively coupled plasma (ICP) dry etching [9,10]. The depth of wet-etched quartz can reach 500 μm [11], but because of the anisotropy of quartz, the sidewall crystal edges can only be reduced through continuous experiments, and cannot be eliminated [12,13,14]. Because wet etching requires a specific orientation of quartz crystal, z-cut quartz is often used for wet etching. According to the relationship between the cutting angle of the quartz crystal and the frequency–temperature characteristics, it is difficult to find the zero-temperature coefficient point of z-cut quartz within its working temperature range. In this case, the large temperature hysteresis of the sensors causes an additional complicated compensation. There are always some challenges when etching delicate and complex structures with different thicknesses using wet etching. Moreover, wet etching is not an environmentally friendly method. LMP-etched quartz ranges in depth from a few microns to hundreds of microns [15,16]. The local high temperature of LMP may cause the quartz to lose its piezoelectric activity, which is quite detrimental, yet the width of the high-temperature areas (over 15 μm) is difficult to narrow. Furthermore, the quality factor, single frequency, and aging rate of quartz resonators are largely determined by the smoothness, parallelism, and geometric correctness of the quartz’s surface. 

Inductively coupled plasma dry etching has proved effective for processing single-crystal quartz. The plasma–chemical etching process can etch a single-crystal quartz plate (z-cut) with a thickness of 369 μm through windows with large linear dimensions (3 mm × 10 mm) [17]. A few institutions have used ICP systems to achieve a depth of quartz of over 100 µm [18,19]. However, small-sized structures are difficult to process in this way and have poor sidewall morphologies. Additionally, the typical single-quartz etching depth (with better morphology) is generally less than 50 μm [20,21,22]. Currently, the thickness of the single-crystal quartz wafer is generally more than 100 μm. Furthermore, considering the increasing fragmentation rate, the processing cost of a thinner quartz wafer will increase. Therefore, it is currently difficult to directly obtain sensors and actuators of crystal quartz using dry etching. The research on the combination of the bonding, thinning, and dry etching of single-crystal quartz in this study aimed to solve these problems by taking advantage of dry etching while avoiding its disadvantages to obtain a favorable device morphology.

## 2. Experimental

### 2.1. The Design of the Overall Process

**Figure 1:**
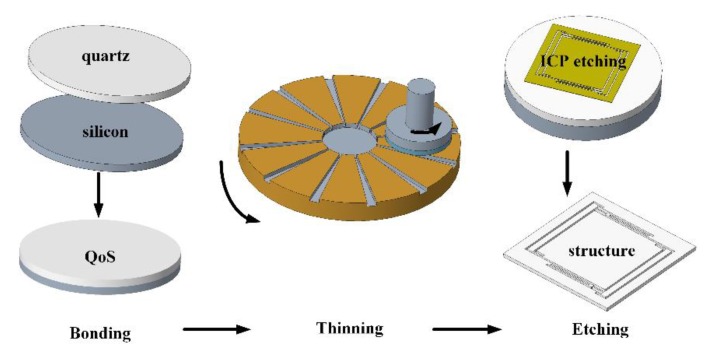
Schematic of the main fabrication process.

In seeking to fabricate a complex three-dimensional structure of single-crystal quartz with a good surface morphology, a novel processing method was proposed that covers the bonding of quartz on silicon (QoS), the thinning and polishing of QoS, and the ICP dry etching of quartz, as shown in Figure 1. Not only was the optimized single-step procedure shown to achieve good results via a sequence of experiments, but the three-step process also proved capable of achieveing excellent compatibility. 

### 2.2. Bonding of Quartz and Silicon

The silicon wafer was bonded with the quartz wafer via direct bonding or auxiliary bonding to obtain the QoS wafer. The original wafers selected for bonding should have a good thickness uniformity and a low surface roughness. Before bonding, the silicon and quartz wafers should be cleaned ultrasonically using acetone and alcohol.

#### 2.2.1. Direct Bonding

Direct bonding [23,24] involves the bonding of two polished wafers without any other materials. Mature anodic bonding is a well-known method for the bonding of silicon and glass [25,26], but it involves bonding at elevated temperatures (higher than 300 °C) with the assistance of a strong electrostatic field [27]. Furthermore, there are no sufficiently mobile alkali metal ions in single-crystal quartz to drive through the bonding interface under an external electric field. Because of the difference in the thermal expansion coefficients between silicon and quartz crystal, the internal stress mismatch at high temperatures leads to the bonded wafer’s breakage. Moreover, quartz crystal loses its piezoelectric character because of the high temperature. Therefore, the low-temperature direct bonding of silicon and quartz was implemented in this work.

(1) The direct bonding process of quartz and silicon

**Figure 2:**
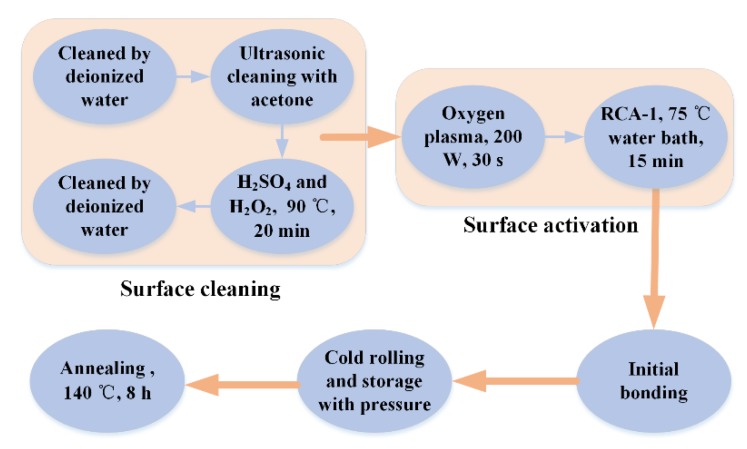
The fabrication process used for direct bonding.

**Figure 3:**
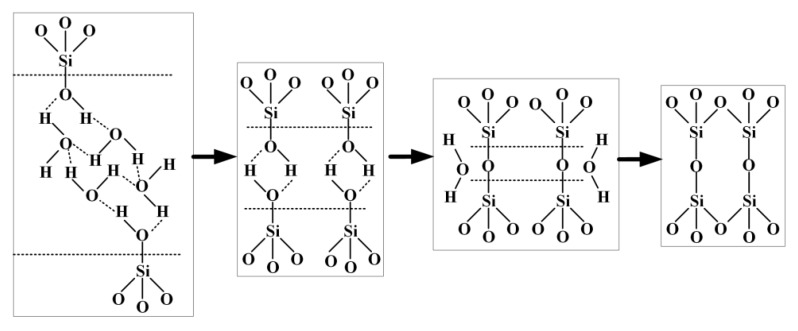
The principle of direct bonding.

The direct bonding procedure entails surface cleaning, surface activation, initial bonding, pressurization, and heat treatment (annealing), as shown in Figure 2. The principle of bonding is Si–OH + Si–OH → Si–O–Si + H_2_O, as shown in Figure 3. A lot of –OH bonds are suspended on the surface of smooth silicon and quartz wafers to generate the surface state of Si–OH. When the two wafers are close enough, intermolecular forces and hydrogen bond forces coordinate to cause the two wafers’ mutual attraction. The Si–OH bond attached to the surface of the wafers is dehydrated to form the Si–O–Si bond [28,29]. Thus, the weak intermolecular force is transformed into the strong force of a covalent bond such that the two bonding wafers are firmly bonded together.

**Figure 4:**
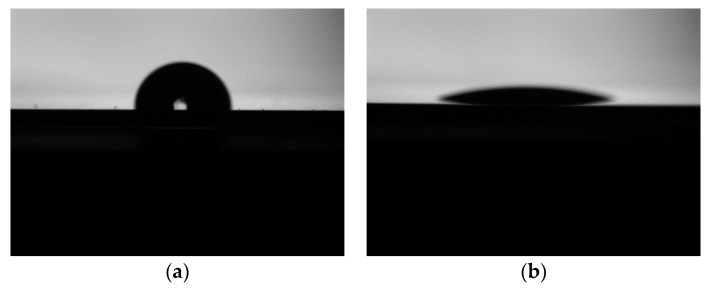
The contact angle of the quartz surface: (**a**) before the quartz surface was activated and (**b**) after the quartz surface was activated.

The first step was the surface cleaning. Two-inch, 400-µm thick silicon wafers (silicon, RDMICRO, Suzhou, China) and 34 mm × 30 mm, 100 μm thick z-cut quartz wafers (quartz, LINDE, Xi’an, China) were selected for direct bonding, where wafers were polished on both sides. Almost all organic and inorganic contaminants on the substrate were completely removed through a series of cleaning steps, which consisted of concentrated sulfuric acid and hydrogen peroxide water bath cleaning, as well as acetone and alcohol ultrasonic cleaning. The second step was the surface activation. Dry activation was implemented first on the surfaces of the quartz and silicon, followed by wet activation. Additionally, the surface of the treated wafers contained hydroxyl groups and was highly hydrophilic, as indicated in Figure 4. Atomic force microscopy (AFM) measurements indicated that the wafer surface treated using dry and wet activation had a root mean square (rms) surface roughness ranging from 0.2 to 0.3 nm.

The third step was the initial bonding. First, the activated wafers were initially aligned and bonded in deionized water for preliminary pre-bonding. Then, a cold rolling pressure of 1 MPa in air was externally exerted, thereby removing the unbonded area’s gas and water, and ensuring that the two wafers bonded more tightly. The pre-bonding wafer was pressurized and stored in air for 8 h to finalize the pre-bonding process. 

The final step was the heat treatment (annealing). The bonding wafer was placed in a programmed oven for low-temperature annealing. The temperature was raised to 140 °C with a gradient of 1 °C/min and was maintained for 8 h. 

(2) The detection of the direct bonding effect

After the process of bonding the two wafers, the bonding quality was evaluated. The testing criteria mainly included the size of the bonding area, the bonding interface, and the bonding strength.

**Figure 5:**
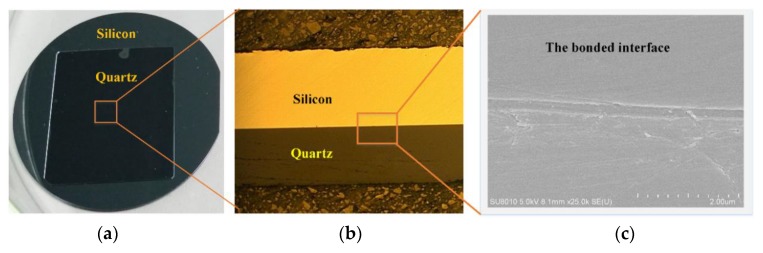
Photograph of quartz on silicon (QoS) via direct bonding: (**a**) the bonded wafer of quartz and silicon, (**b**) optical microscopy image of the cross-sectional view of QoS, and (**c**) SEM photograph of the cross-sectional view of QoS.

The size of the bonding area: Because of the transparency of quartz material, the bonding area of QoS could be observed via visual inspection, which is shown in Figure 5a. The quartz and silicon were largely bonded, but a small part of the edge of the quartz could not be bonded completely because of a poor edge flatness and the few inevitable contaminants that arose in the operating environment.

The bonding interface: The interface was inspected using an optical microscope and scanning electron microscope (SEM), as shown in Figure 5b,c. The images show that the interface of QoS was completely bonded without any defects.

**Figure 6:**
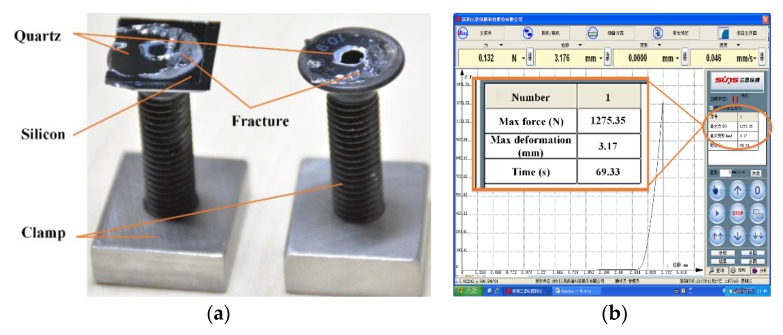
Tensile test of the silicon–quartz bonded wafer: (**a**) the fracture surface of the broken QoS wafer after stretching and (**b**) the tensile force of the bonded wafer.

The bonding strength: To further detect the bonding quality, the bonded wafer was fixed onto a testing machine using a specific fixture to test the tensile strength. It can be seen from Figure 6a that the fractures mainly occurred inside the quartz material after stretching, and partial fractures appeared in the bonding glue; slight partial fractures occurred in the bonding interface, which indicated that the bonding strength was high, but the bonding uniformity was somewhat poor. It can be seen from Figure 6b that the axial force (F) was 1273.35 N. The bonding area (S) was approximately 19.6 × 16.8 mm = 329.28 mm^2^. Substituting the data into σ = F/S, yields a tensile strength (σ) of 3.8 MPa, which was the bonding strength.

Subsequently, the bonding wafers were immersed in water, alcohol, and acetone in sequence for more than 8 h, tested using ultrasonic cleaning, and bombarded with plasma for a long time. The interface between both bonding wafers was well retained without any reduction in the bonded surface. However, the bonding process of quartz and silicon was not mature enough, and its low yield was not conducive to the continued research of the subsequent process. Therefore, auxiliary bonding was proposed for continued research.

#### 2.2.2. Auxiliary Bonding

Auxiliary bonding is the bonding of two homogenous or heterogeneous materials with the assistance of a third material. Common auxiliary bonding methods include the adhesive method, soldering and brazing, low-melting glass paste, and laser thermal fusion welding. Because of its lower cost, simplicity, compatibility with subsequent processes, and better removal of silicon wafers, the adhesive method was selected to research the subsequent process.

(1) The auxiliary bonding process.

Adhesive bonding is the bonding of silicon and quartz wafers using epoxy resin adhesive (CY1003, SIRNICE, Guangzhou, China). Two-inch, 500-µm thick silicon wafers (silicon, RDMICRO, Suzhou, China) and 300-μm thick Z-cut quartz wafers (quartz, RDMICRO, Suzhou, China) were selected for auxiliary bonding, where both wafers were polished on both sides. The quartz and silicon wafers were required to have good total thickness variation (TTV) and sufficiently clean surfaces without any contaminants. To move forward to the following processes, auxiliary bonding has strict requirements for glue, such as low viscosity, low shrinkage, high-temperature resistance, resistance to acetone corrosion, and good thermal conductivity. 

The glue must have a low viscosity to ensure that it is evenly and thinly applied to the surface of the wafer. The viscosity of the glue should be under 5000 mPa·s. Here, glue with a viscosity of 1500 mPa·s was selected. A curing time of 1–2 h was suitable. The choice of curing time should be sufficient to evacuate and apply pressure while avoiding a reduction efficiency due to too long of a curing time. 

The method used for curing the glue should be room-temperature curing or ultraviolet curing (because quartz material is transparent). After the experiments, when the tensile strength was greater than 0.8 MPa and the shear strength was greater than 0.5 MPa, the bonded wafers could be used for thinning and polishing without de-bonding or fragmenting.

**Figure 7:**
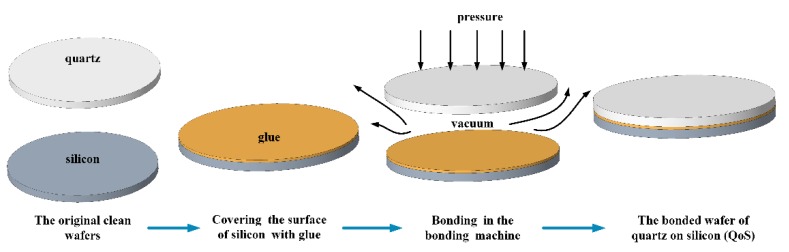
The fabrication process of auxiliary bonding.

The bonding process is shown in Figure 7. In an ultra-clean room, the bonding glue was evenly spun onto the silicon using a spin coater with a low speed of 500 r/min for 9 s and a high speed of 4000 r/min for 40 s. The silicon wafer was placed in a special fixture with the glue facing up. The quartz wafer was placed on the silicon wafer, aligned with the silicon wafer, and placed in a vacuum press-bonding machine (machine type: TWB-100, RDMICRO, Suzhou, China; the maximum pressure was 7 bar and the pressure uniformity was ±5%). Then, the bonder was sealed. The bonding temperature was set to 30 °C. The vacuum was set to 0.1 mbar. The bonding pressure was set to 2 bar. Then, the device was turned on for automatic bonding. The bonding time was kept for 2 h (according to the curing time of the glue). Then, the bonded wafer was removed from the bonder.

(2) The detection of auxiliary bonding

**Figure 8:**
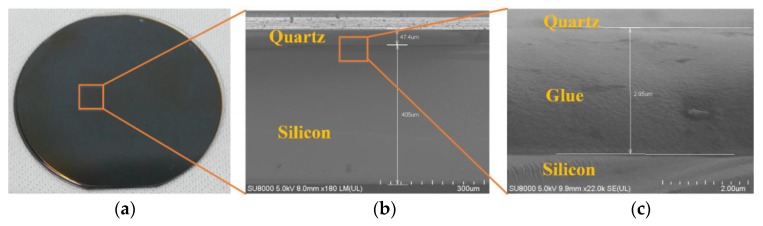
Images of QoS using auxiliary bonding: (**a**) the bonded wafer of quartz and silicon, (**b**) SEM photograph of the cross-sectional view of QoS, and (**c**) partial enlargement view of the SEM photograph.

After a couple of experiments, in addition to determining the appropriate glue, a perfectly bonded wafer was acquired, as shown in Figure 8. It can be inferred from the SEM images (zoom-in of the cross-section at the bonding interface) that the bonding uniformity achieved the desired result. The bonding strength was ensured by the adhesive strength of the glue itself. According to the performance parameters of the glue, its tensile strength and shear strength were greater than 0.8 MPa and 0.5 MPa, respectively.

### 2.3. Thinning and Dry Etching of Quartz

#### 2.3.1. The Thinning of Quartz

By reducing the thickness of the quartz, the quartz wafer could be etched through directly from one side. There were four steps used for thinning the quartz: bonding, thinning, polishing, and cleaning of the QoS wafer.

QoS with a good TTV (<10 μm) was selected for this experiment. The QoS wafer was bonded to the glass substrate using wax. The wax was easily controlled to maintain a uniform thickness of the bonding between the QoS and glass substrate and was conveniently cleaned from the QoS wafer. Then, after the QoS was bonded with the glass substrate, the TTV was measured again. If the flatness of the bonded wafer is in the range of several microns after being glued to the glass substrate, then it was suitable for grinding and polishing. If the flatness difference was too large (greater than 10 microns), then the bonded wafer needed to be re-bonded.

**Figure 9:**
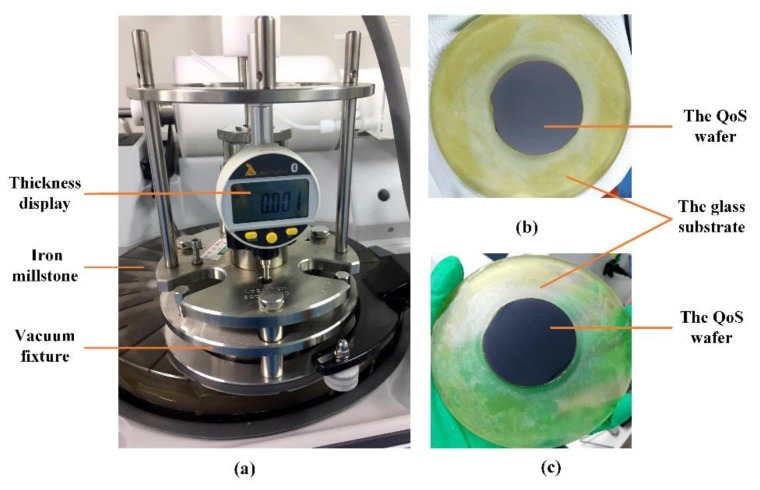
The thinning and polishing of the QoS wafer on the precision lapping and polishing machine: (**a**) the precision lapping and polishing machine, (**b**) QoS after lapping, and (**c**) QoS after polishing.

Then, the QoS wafer was lapped and polished on a precision lapping machine (PM5, Logitech, UK), as shown in Figure 9. The speed and quality of the thinning were controlled by controlling the dripping speed of the slurry, the rotational speed of the turntable, and the pressure of the grinding. When the thickness and surface roughness of quartz were 40 μm (or another required thickness) and less than 5 nm, respectively, the entire process of thinning and polishing was completed.

Afterward, a qualified QoS was obtained. The QoS was taken from the glass substrate to melt the wax via heating. Then, the QoS was immersed in a special de-waxing liquid at a temperature of 120 °C for 10–20 min to remove the residual wax from the surface of the QoS. Then, the QoS was washed using acetone, alcohol, and deionized water. Thus, the QoS wafer was prepared for the next step.

#### 2.3.2. The ICP Dry Etching of Quartz

The ICP dry etching of quartz was a huge challenge because of its hardness characteristic of quartz. The ICP dry etching research in this paper mainly focused on study of the etching mask and etching gas. The desired etching result was achieved by the selection of materials and thickness of the structure’s masks, the composition and ratio of the etching gas, the etching power, and the chamber temperature.

(1) The quartz-structure etching mask.

**Figure 10:**
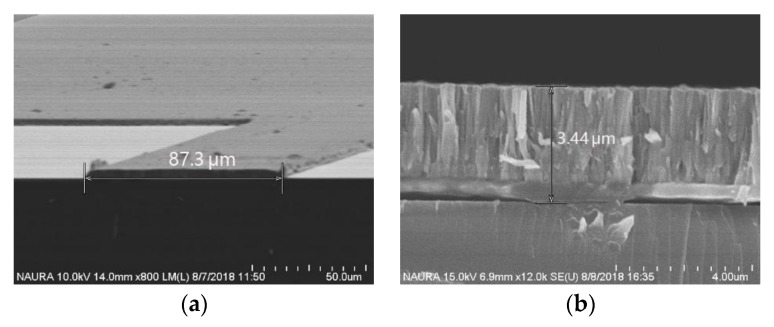
The quartz structure etching mask of Cr: (**a**) SEM photograph of the top view of the mask and (**b**) SEM photograph of the cross-sectional view of the mask.

After comparing the etching effects of a series of materials, such as aluminum, nickel, chromium, and a special photoresist, especially the selection ration, chromium was selected as the etching mask. The quartz structure etching mask of Cr was deposited using a standard micro-electromechanical systems (MEMS) process with a thickness of more than 3 μm, as shown in Figure 10. Because the mask was too thick, it was sputtered in a “sandwich” form to increase the adhesion between the mask and the substrate and to release the internal stress of sputtering. That is, the “sandwich” mask was alternately composed of chromium and aluminum. Moreover, the material of the uppermost layer of the mask was aluminum oxide to increase the etching selectivity ratio.

(2) The ICP etching process of quartz

The fact that a micro quartz structure was obtained using ICP dry etching proved the rationality of the processing method. The etching process was continuously adjusted using various ratios of the etching gas, chamber temperature, and bias power to obtain vertical sidewalls, a small roughness, a high selectivity ratio, and a high etching rate [30]. 

**Figure 11:**
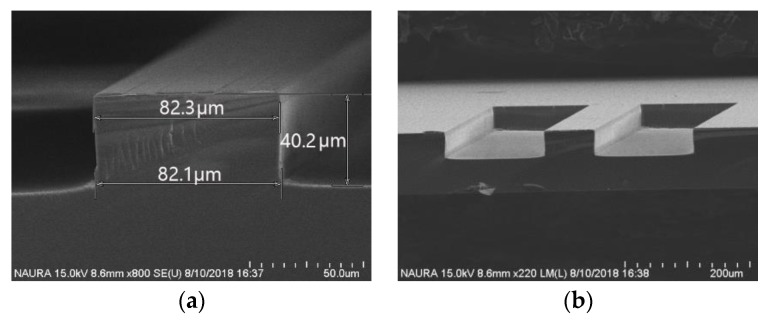
SEM image of the quartz etching structure: (**a**) SEM photograph of the cross-sectional view of the etching structure and (**b**) SEM photograph of the overall view of the etching structure.

After a sequence of experiments, the experimental parameters were selected: CF_4_ etching gas with flow rate of 100 sccm, etching temperature of 80 °C, Radio Frequency (RF) power of 1200 W, and a bias power of 120 W. Eventually, the etching depth was above 40 μm, the etching selectivity was above 13, and the etching rate was above 280 nm/min, as shown in Figure 11. 

## 3. Results and Discussion

**Figure 12:**
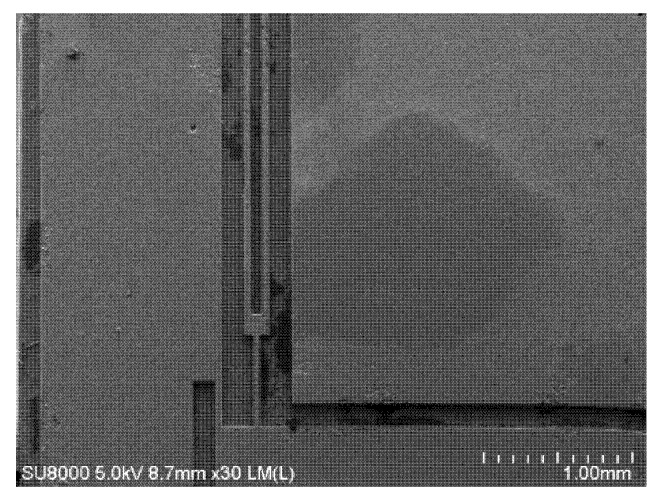
SEM photograph of the top view of the quartz etching structure.

After the etched quartz structure was obtained, the auxiliary layer of silicon was removed to obtain a structure of pure quartz, as shown in Figure 12, or the silicon layer was further processed to obtain a composite structure containing quartz and silicon materials.

In the final stage of removing the silicon wafer, the adhesive glue layer in the middle of the QoS (obtained via auxiliary bonding) also needed to be removed. This problem could be avoided if direct bonding was substituted for auxiliary bonding. In addition, direct bonding, without the participation of a third material, is more conducive to controlling the TTV of the bonded wafer, which is conducive to the subsequent thinning, polishing, and dry etching of quartz. In the future, the auxiliary bonding will be replaced with direct bonding to complete the entire process.

## 4. Conclusions

A new processing technique, including the bonding of quartz and silicon, as well as the thinning and dry etching of quartz, was introduced and proven to be feasible. With the assistance of the silicon layer, ultra-thin quartz materials were obtained and complex three-dimensional quartz structures with a good morphology and steep sidewalls were obtained by using this process. Then, the structure of the pure quartz material was obtained by removing the silicon wafer. Compared with the separated structure, the integrated structure has many advantages. The single material is beneficial for improving the performance of the device without inconsistent thermal expansion coefficients or assembly error. Without the problem of the thermal expansion coefficient, the effect of temperature on the device’s performance can be sufficiently reduced. The glue caused neither moisture or an aging problem during assembly. In addition, the silicon layer was processed into the appropriate structure. A similar method could also be introduced to process other quartz-based sensors and actuators. Moreover, it provides a feasible idea for fabricating multilayer structures of heterogeneous materials.
